# Metabolomics reveals high fructose-1,6-bisphosphate from fluoride-resistant *Streptococcus mutans*

**DOI:** 10.1186/s12866-024-03310-8

**Published:** 2024-05-03

**Authors:** Laikuan Zhu, Jiehang Li, Yueping Pan, Jing Huang, Hui Yao

**Affiliations:** 1 Department of Endodontics and Operative Dentistry, Shanghai Ninth People’s Hospital, Shanghai Jiao Tong University School of Medicine, 639 Zhizaoju Road, Shanghai, 200011 China; 2 College of Stomatology, National Center for Stomatology, National Clinical Research Center for Oral Diseases, Shanghai Key Laboratory of Stomatology, Shanghai Jiao Tong University, Shanghai Research Institute of Stomatology, Shanghai, 200011 China; 3 Department of Stomatology, Hainan Western Central Hospital, Hainan, 571700 China; 4 Department of Oral Medicine, Shanghai Ninth People’s Hospital, Shanghai Jiao Tong University School of Medicine, 639 Zhizaoju Road, Shanghai, 200011 China

**Keywords:** *Streptococcus mutans*, Fluoride resistance, Metabolomics, Fructose-1,6-bisphosphate, Dental caries, Mass spectrometry

## Abstract

**Background:**

Fluoride-resistant *Streptococcus mutans* (*S. mutans*) strains have developed due to the wide use of fluoride in dental caries prevention. However, the metabolomics of fluoride-resistant *S. mutans* remains unclear.

**Objective:**

This study aimed to identify metabolites that discriminate fluoride-resistant from wild-type *S. mutans*.

**Materials and methods:**

Cell supernatants from fluoride-resistant and wild-type *S. mutans* were collected and analyzed by liquid chromatography-mass spectrometry. Principal components analysis and partial least-squares discriminant analysis were performed for the statistical analysis by variable influence on projection (VIP > 2.0) and *p* value (Mann–Whitney test, *p* < 0.05). Metabolites were assessed qualitatively using the Human Metabolome Database version 2.0 (http://www.hmdb.ca), or Kyoto Encyclopedia of Genes and Genomes (http://www.kegg.jp), and Metaboanalyst 6.0 (https://www.metaboanalyst.ca).

**Results:**

Fourteen metabolites differed significantly between fluoride-resistant and wild-type strains in the early log phase. Among these metabolites, 5 were identified. There were 32 differential metabolites between the two strains in the stationary phase, 13 of which were identified. The pyrimidine metabolism for *S. mutans* FR was matched with the metabolic pathway.

**Conclusions:**

The fructose-1,6-bisphosphate concentration increased in fluoride-resistant strains under acidic conditions, suggesting enhanced acidogenicity and acid tolerance. This metabolite may be a promising target for elucidating the cariogenic and fluoride resistant mechanisms of *S. mutans*.

**Supplementary Information:**

The online version contains supplementary material available at 10.1186/s12866-024-03310-8.

## Background

*Streptococcus mutans* (*S. mutans*) is a major cariogenic pathogen, known as a predominant etiological contributor to dental caries [1, 2]. Its virulence factors are involved in the acidogenicity, the acid tolerance, and the biofilm formation [3]. Currently, fluoride is one of the most representative caries-preventive agents through reducing the hydroxyapatite solubility. Fluoride can depress demineralization and enhance remineralization of the dental enamels [4]. Fluoride is also proved to inhibit the *S. mutans* metabolism [5]. Nevertheless, fluoride-resistant *S. mutans* (*S. mutans* FR) appears with the extensive, long-lasting application of fluoride. *S. mutans* FR obtains the phenotype of enhanced acidogenicity and acid tolerance due to the gene alteration of *eriC*^*F*^, encoding fluoride antiporters [6–8]. Since the enamel demineralization prevention effects are reduced, fluoride cannot effectively inhibit *S. mutans* FR [9–11].

Until now, the specific mechanism remains unclear, by which the fluoride-resistant strains are more powerful in acidogenicity and acid tolerance than the wild-type ones. In recent years, metabolomics focusing on the quantitative analysis of small molecules with < 1,000 molecular weight [12], which may play critical roles in the metabolic processes [13], allows us to analyze the metabolomes of target cells with their phenotypic difference, thereby to better explore the underlying mechanism of their difference [14]. The mass spectrometry (MS) is an indispensable tool for metabolomic analysis, especially when combined with liquid chromatography (LC).

Previously, molecular changes and carbohydrate metabolism were demonstrated during the development of dental caries. For example, alteration of 37 metabolites was confirmed by MS when culturing *S. mutans* at different pH values [15]. Furthermore, *S. mutans* biofilm growth [16, 17] and acid production [17] were affected by carbohydrate metabolism. *S. mutans* membrane vesicles enhanced the metabolite expression of *Candida albicans* related to carbohydrate metabolism [18]. In the carbohydrate metabolism process, when fluoride was added into the caries-associated bacteria, for instance, *Scardovia wiggsiae*, the pyruvate level decreases [19]. *Bifidobacterium* could metabolize carbohydrates and produce acid by the bifid shunt even in the presence of fluoride [20]. Metabolomic effects of fluoride on plaque biofilm i*n vivo* revealed fluoride inhibited acid production, along with the phosphoenolpyruvate decrease in the Embden-Meyerhof-Parnas pathway [21].

However, metabolomic data on *S. mutans* FR are rare. Discriminant metabolomes between fluoride-resistant strains and wild-type ones have not been identified yet. Herein, we did a metabolic analysis of cells from the two *S. mutans* strains by LC-MS technique. The study aims to determine whether marked variations in the metabolome of the two *S. mutans* strains exist.

## Materials and methods

### Bacteria and growth conditions

As in our previous study [22], we grew wild-type *S. mutans* (UA159, also named *S. mutans* UA), stored in a 50% (v/v) glycerol solution at − 80°C in brain heart infusion (BHI) broth (BHIB; Difco) or on BHI agar (BHIA; Difco) under anaerobic conditions (37°C, 95% N_2_, 5% CO_2_). Then we sub-cultured UA159 on BHIA and obtained fluoride-resistant *S. mutans* (UA-FR, also named *S. mutans* FR) through increasing the sodium-fluoride (NaF) concentrations from 50 µg/mL to 1,000 µg/mL gradually. Each *S. mutans* FR isolate in this study was selected at a NaF concentration of 1,000 µg/mL.

### Sample collection and preparation

UA159 and UA-FR in suspension were anaerobically cultured in BHIB containing 1% glucose at 37°C through entering the early or the stationary stage of bacterial growth. The metabolic activities of the bacteria ceased when 5 mL broth was mixed with 10 mL 60% methanol aqueous solution at − 48°C. The clarified supernatants were discarded after centrifugation at 4500 r/min for 10 min at − 19°C.

Bacteria were harvested under such conditions after washing twice. A total of 500 µL 100% methanol (− 48°C) was added to the bacteria. The mixture was frozen in liquid nitrogen and thawed on ice. After three freeze-thaw cycles, the mixture was centrifugated at 16,000 ×*g* for 5 min at − 19°C and the first supernatants were collected. After the centrifugation, the remaining bacteria were mixed again with 500 µL 100% methanol (− 48°C). The freeze-thaw cycles and centrifugation were repeated. The second supernatants were collected. Finally, the second supernatants were mixed with the first ones. The mixed supernatants were stored at − 80°C for metabolomic analysis using liquid chromatography coupled with mass spectrometry (LC-MS).

### LC-MS analysis

The supernatants were analyzed by a liquid chromatography column (Agilent) coupled with a micrOTOF-Q II mass spectrometer (Bruker). The mobile phase included 0.1% formic acid solution (A) and acetonitrile (B). The flow rate was 0.35 mL/min. Initially, 2% acetonitrile was maintained for 0.5 min. Then, the acetonitrile concentration was linearly increased to 100% within 24 min and was maintained for 3.5 min. After that, the acetonitrile concentration was reduced back to 2%. The column was equilibrated for 2 min and the column temperature was 35°C. The temperature of the automatic sampler was maintained at 4°C. The sample load was 2 µL.

The MS conditions were set as follows: source mode, both positive and negative modes of electrospray ionization (ESI); mass scanning range, 100 − 1,000 m/z; spray gas flow rate, 1.5 L/min; dry gas pressure, 0.2 MPa; heating temperature, 200°C; ion accumulation time, 20 ms; detection voltage, 1.6 kV; interface voltage, 4.5 kV and − 3.5 kV for the positive and the negative poles, respectively. The acquisition parameters are shown in Table 1.

**Table 1:** Acquisition parameters of LC-MS

Parameter	Value
**LC**	
Mobile phase	0.1% formic acid solution (A) and acetonitrile (B) with a gradient elution as follows: 0–0.5 min, 2% B; 0.5–24.5 min, 2–100% B; 24.5–28 min, 100% B; 28–30 min, 2% B;
Flow rate	0.35 mL/min
Column temperature	35 °C
Autosampler temperature	4 °C
Load volume	2 µL
**MS**	
Source type	Electrospray ionization
Ion polarity	Positive and negative
Interface voltage	4.5 kV and − 3.5 kV
Mass scanning range	100–1,000 m/z
Dry gas pressure	0.2 MPa
Ion accumulation time	20 ms
Detection voltage	1.6 kV
Heating temperature	200 °C
Gas flow rate	1.5 L/min

The two isolates (*S. mutans* UA and *S. mutans* FR) in the two phases (in early log phase and in stationary phase) were used via the two LC-MS TIC chromatograms (RP and HILIC) in this study.

### Statistical analysis

The raw data collected using the LC-MS device were proceeded with peak extraction and peak matching. Peak discrimination, peak alignment, total peak area normalization, data scaling, and missing value removal by the correction of 80% rule were also performed. After the proceeded data were normalized by peak area, the potential discriminant metabolites were matched with entries in the version 2.0 of Human Metabolome Database (HMDB, http://www.hmdb.ca), or Kyoto Encyclopedia of Genes and Genomes (KEGG, http://www.kegg.jp). Open database source (Metaboanalyst 6.0, https://www.metaboanalyst.ca) was applied to identify metabolic pathways.

Orthogonal signal correction (OSC) method was applied to screen out part of the group-independent matrices. The three-dimensional matrix obtained was analyzed by SIMCA-P 12.0 software (Umetrics). The aggregation, dispersion and outliers of the samples were assessed by principal component analysis (PCA) in the filtered matrix. Subsequently, partial least-squares discriminant analysis (PLS-DA) was used to identify the major variations that contributed to this aggregation and dispersion. The discriminant metabolites were determined in the single-dimensional statistics by variable influence on projection (VIP > 2.0) and *p* value (Mann–Whitney test, *p* < 0.05). The quality of the PCA and PLS-DA was assessed by the parameters R^2^ and Q^2^.

## Results

**Fig. 1:**
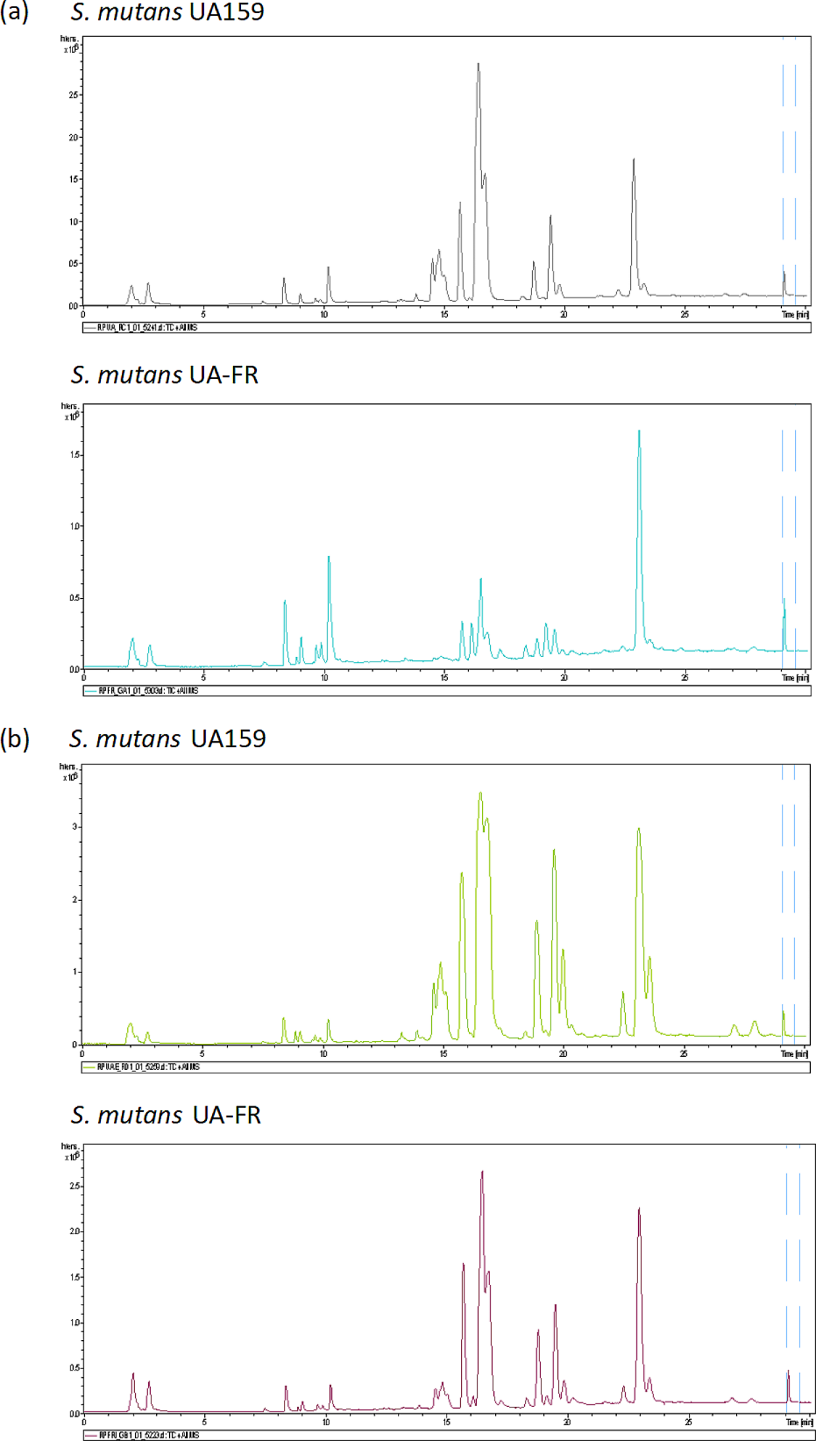
Typical RP LC-MS TIC chromatograms of *S. mutans*. (a) in early log phase; (b) in stationary phase. RP, reversed phase; LC-MS, liquid chromatography-mass spectrometry; TIC, total ion current; *S. mutans, Streptococcus mutans. S. mutans* UA159 represents wild-type *S. mutans. S. mutans* UA-FR represents fluoride-resistant *S. mutans*

**Fig. 2:**
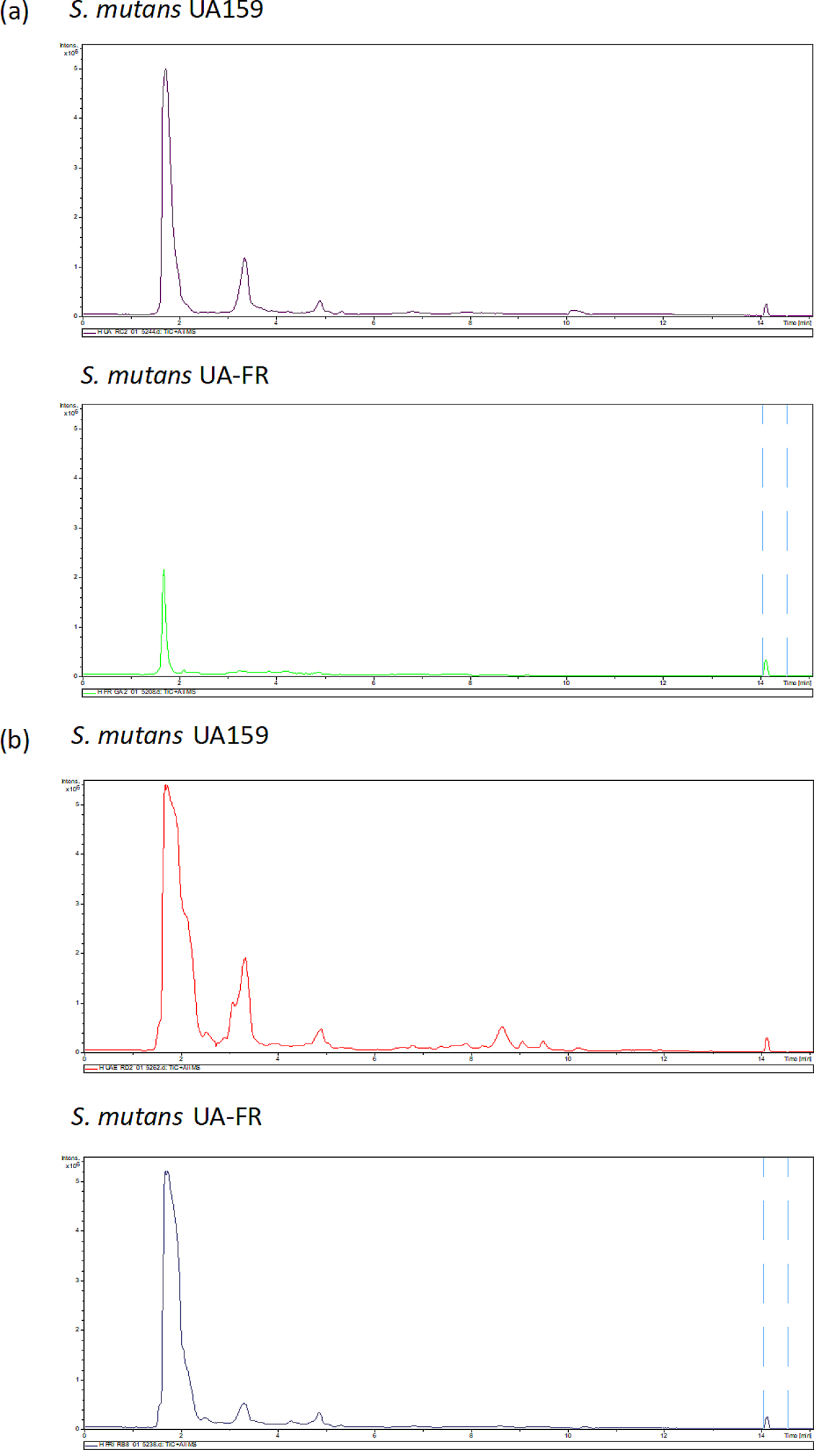
Typical HILIC LC-MS TIC chromatograms of *S. mutans*. (a) in early log phase; (b) in stationary phase. HILIC, hydrophilic interaction liquid chromatography; LC-MS, liquid chromatography-mass spectrometry; TIC, total ion current; *S. mutans, Streptococcus mutans. S. mutans* UA159 represents wild-type *S. mutans. S. mutans* UA-FR represents fluoride-resistant *S. mutans*

The reversed phase (RP) LC-MS total ion current (TIC) chromatograms of the cells from *S. mutans* UA and *S. mutans* FR in early log phase and in stationary phase are shown in Fig. 1, where significant differences of some spectrum peaks existed between the two *S. mutans* strains. For example, in the RP LC-MS TIC chromatograms, most peaks of *S. mutans* FR in early log phase were higher than those of *S. mutans* UA in early log phase (e.g. peaks at retention time of about 8 − 10 and 23 min in Fig. 1a), while some peaks of *S. mutans* FR in early log phase were lower (e.g. peaks at retention time of 15–20 min in Fig. 1a). At retention time of 15–20 min, similarly, the peaks of *S. mutans* FR in stationary phase were also lower (Fig. 1b). Remarkable variations between them were also demonstrated in the hydrophilic interaction liquid chromatography (HILIC) LC-MS TIC chromatograms (Fig. 2). Complementary to the RP method, the HILIC one was suitable to retain polar metabolites. At retention time of about 2 and 3 min, the peaks of *S. mutans* FR were lower than those of *S. mutans* UA both in early log phase (Fig. 2a) and in stationary phase (Fig. 2b).

**Fig. 3:**
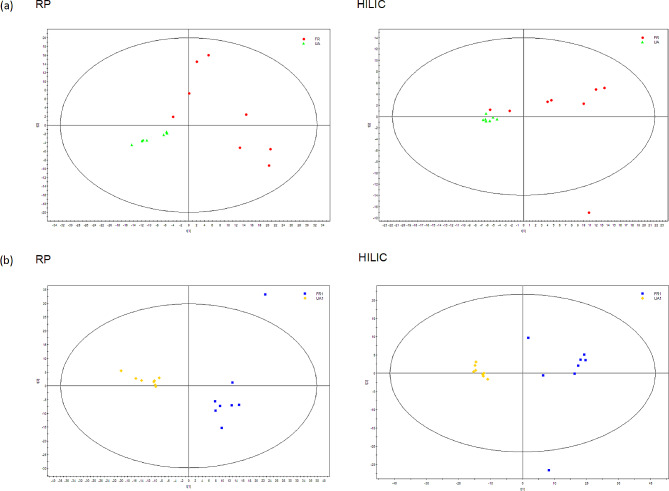
PCA scores plot of LC-MS spectral data on *S. mutans.* (a) in early log phase; (b) in stationary phase. PCA, principal component analysis; LC-MS, liquid chromatography-mass spectrometry; *S. mutans, Streptococcus mutans;* RP, reversed phase; HILIC, hydrophilic interaction liquid chromatography. UA represents wild-type (UA159) *S. mutans.* FR represents fluoride-resistant *S. mutans*

**Fig. 4:**
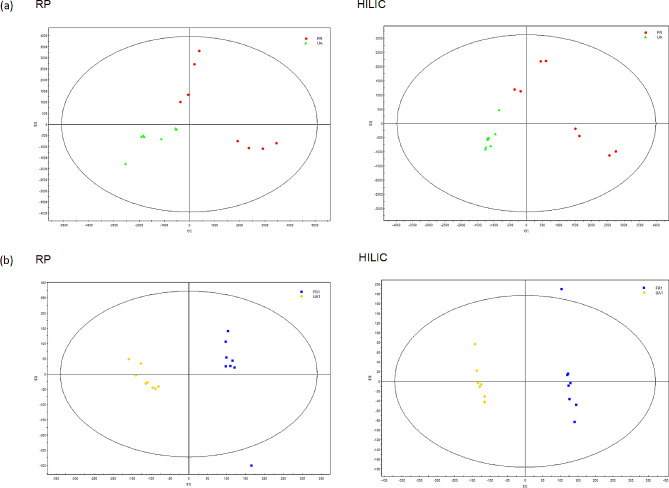
PLS-DA scores plot of LC-MS spectral data on *S. mutans.* (a) in early log phase; (b) in stationary phase. PLS-DA, partial least squares discriminant analysis; LC-MS, liquid chromatography-mass spectrometry; *S. mutans, Streptococcus mutans;* RP, reversed phase; HILIC, hydrophilic interaction liquid chromatography. UA represents wild-type (UA159) *S. mutans.* FR represents fluoride-resistant *S. mutans*

After data normalization, including the removal of internal standards and pseudo-positive peaks and the combination of the same metabolite peaks, the PCA was used in the data analysis and the scores plot demonstrated a trend separating between the two *S. mutans* strains (Fig. 3). The supervised PLS-DA (Fig. 4) was performed on the dataset, also showing two separated clusters (*S. mutans* UA and *S. mutans* FR). When constructing the PCA model, the SIMCA-P software typically segmented the data into seven groups sequentially. Six of these groups were employed to establish a foundational model, which served as the basis for predicting the accuracy of the remaining group. This process was iterated several times to derive the Q^2^ value. Such cross-validation in SIMCA-P was consistently employed to ascertain the optimal number of principal components and to mitigate the risk of overfitting. The modeling diagnostic confirming the validity of PCA and PLS-DA model is shown in Table S1,2. The model validation assured that this PLS-DA model was reliable in interpreting and predicting the variations in either the RP column (R^2^Y = 0.784 and Q^2^Y = 0.719) or the HILIC column (R^2^Y = 0.891 and Q^2^Y = 0.793) (Table S1) during early log phase. This validation also confirmed the robustness of the PLS-DA model in the RP column (R^2^Y = 0.492 and Q^2^Y = 0.405) and the HILIC column (R^2^Y = 0.499 and Q^2^Y = 0.425) during stationary phase (Table S2).

The loading plot (Fig. S1) was performed to assess the marked metabolites through the weightings on the distinguished clusters. In the loading plot, each point represented a metabolite, and the greater the distance from the major ion clusters, the more significant the metabolite’s contribution to sample category separation, signifying its potential as a biomarker. The clustered portion comprised metabolites common to both groups, contributing minimally to sample category separation. Conversely, substances with a scattered distribution contributed significantly to category separation. The S-plot was applied in the selection of the discriminant metabolites differently expressed between the two *S. mutans* strains (Fig. S2). In the S-plot, each point represented a variable, indicating the correlation between variables and the model. Some variables far from the origin contributed significantly to differentiating the clustering between two groups. Other variables with small correlation coefficients and large confidence intervals were excluded. Variables with VIP values exceeding 2.0, marked by red boxes, were selected as potential characteristic metabolites of *S. mutans* FR. These metabolites provided a basis for exploring the physiological properties of *S. mutans* FR.

After the PLS-DA analysis of the two *S. mutans* strains in early log phase, a total of 14 differential metabolites with VIP > 2.0 and *p* < 0.05 (Mann–Whitney test) in one-dimensional statistical analysis were identified as potential metabolic markers. Among these 14 differential metabolites (*n* = 9, hydrophobic metabolites; *n* = 5, hydrophilic metabolites) of *S. mutans* FR, 3 were elevated and 11 were reduced at concentration compared to *S. mutans* UA (5 identified markers shown in Table 2).

**Table 2:** Comparison of metabolites between the fluoride-resistant strain and the wild-type strain in early log phase

Metabolite	Chemical formula	Rt RP (s)^†^	Rt HILIC (s)^‡^	m/z	VIP^§^	Trend^¶^	Metabolic pathway	HMDB ID	KEGG ID
1-octadecanoyl-sn-glycerol 3-phosphate	C_21_H_41_O_7_P_1_	613.1	-	437	3.19	↑	Fatty acid metabolism	HMDB07854	-
6-phospho-D-glucono-1,5-lactone	C_6_H_9_O_9_P_1_	942.0	-	256	7.22	↓	Carbohydrate metabolism	HMDB01127	C01236
CMP	C_9_H_12_N_3_O_8_P_1_	-	140.1	321	2.34	↓	Nucleotide metabolism	HMDB00095	C00055
Deoxycytidine	C_9_H_13_N_3_O_4_	546.0	-	227	2.24	↑	Nucleotide metabolism	HMDB00014	C00881
Pyridoxamine 5’-phosphate	C_8_H_12_N_2_O_5_P_1_	-	149.3	247	2.49	↓	Amino acid metabolism	HMDB01555	C00647

^†^Rt RP (s), retention time of reversed phase column (second)

^‡^Rt HILIC (s), retention time of hydrophilic interaction liquid chromatography column (second)

^§^VIP, variable important on projection; VIP was obtained from PLS-DA with a threshold of 2.0

^¶^Trend: The levels of the discriminant metabolites were labeled with (↓) downregulated and (↑) upregulated when the fluoride-resistant strain compared to the wild-type strain (Mann–Whitney test, *p* < 0.05)

In stationary phase, 32 differential metabolites (*n* = 22, hydrophobic metabolites; *n* = 10, hydrophilic metabolites) with VIP > 2.0 and *p* < 0.05 (Mann–Whitney test) were found in the two *S. mutans* strains. At concentration, 14 of *S. mutans* FR were increased and 18 of *S. mutans* FR were reduced among them (13 identified markers shown in Table 3).

**Table 3:** Comparison of metabolites between the fluoride-resistant strain and the wild-type strain in stationary phase

Metabolite	Chemical formula	Rt RP (s)^†^	Rt HILIC (s)^‡^	m/z	VIP^§^	Trend^¶^	Metabolic pathway	HMDB ID	KEGG ID
1-octadecanoyl-sn-glycerol 3-phosphate	C_21_H_41_O_7_P_1_	613.6	-	437	2.21	↑	Fatty acid metabolism	HMDB07854	-
2,3-diphosphoglycerate	C_3_H_3_O_10_P_2_	-	206.7	261	2.66	↑	Carbohydrate metabolism	HMDB01294	C01159
3-keto-L-gulonate 6-phosphate	C_6_H_8_O_10_P_1_	1041.7	-	270	2.29	↓	Carbohydrate metabolism	-	C14899
6-phospho-D-glucono-1,5-lactone	C_6_H_9_O_9_P_1_	-	110.6	256	3.16	↑	Carbohydrate metabolism	HMDB01127	C01236
Aminopropyl cadaverine	C_8_H_24_N_3_	120.0	508.8	162	2.87	↓	Amino acid metabolism	HMDB12189	C16565
CMP	C_9_H_12_N_3_O_8_P_1_	1411.1	-	321	2.82	↓	Nucleotide metabolism	HMDB00095	C00055
dCMP	C_9_H_12_N_3_O_7_P_1_	-	207.8	305	2.19	↑	Nucleotide metabolism	HMDB01202	C00239
D-ribose-5-phosphate	C_5_H_9_O_8_P_1_	794.0	-	228	2.98	↓	Carbohydrate metabolism	HMDB01548	C00117
D-sedoheptulose-1,7-bisphosphate	C_7_H_12_O_13_P_2_	1677.6	-	366	3.26	↓	Carbohydrate metabolism	HMDB60274	-
dUDP	C_9_H_11_N_2_O_11_P_2_	-	400.9	385	2.15	↑	Nucleotide metabolism	HMDB01000	C01346
Fructose-1,6-bisphosphate	C_6_H_10_O_12_P_2_	1221.4	-	336	2.61	↑	Carbohydrate metabolism	HMDB01058	-
GMP	C_10_H_12_N_5_O_8_P_1_	1410.3	-	360	2.67	↓	Nucleotide metabolism	HMDB01397	C00144
Pyridoxamine 5’-phosphate	C_8_H_12_N_2_O_5_P_1_	1042.5	114.6	247	2.03	↑	Amino acid metabolism	HMDB01555	C00647

^†^Rt RP (s), retention time of reversed phase column (second)

^‡^Rt HILIC (s), retention time of hydrophilic interaction liquid chromatography column (second)

^§^VIP, variable important on projection; VIP was obtained from PLS-DA with a threshold of 2.0

^¶^Trend: The levels of the discriminant metabolites were labeled with (↓) downregulated and (↑) upregulated when the fluoride-resistant strain compared to the wild-type strain (Mann–Whitney test, *p* < 0.05)

In early log phase and in stationary phase, the common significant discriminant metabolites were 1-octadecanoyl-sn-glycerol 3-phosphate, 6-phospho-D-glucono-1,5-lactone, CMP, and pyridoxamine 5’-phosphate. Interestingly, reduced 6-phospho-D-glucono-1,5-lactone and pyridoxamine 5’-phosphate levels from *S. mutans* FR were observed when compared to the levels from *S. mutans* UA in early log phase. However, the case was the opposite in the stationary phase. Deoxycytidine was the only discriminant metabolite in early log phase, whereas the 9 discriminant metabolites were 2,3-diphosphoglycerate, 3-keto-L-gulonate 6-phosphate, aminopropyl cadaverine, dCMP, D-ribose-5-phosphate, D-sedoheptulose-1,7-bisphosphate, dUDP, fructose-1,6-bisphosphate, and GMP in stationary phase merely. Notably, in stationary phase, aminopropyl cadaverine and pyridoxamine 5’-phosphate were the 2 discriminant metabolites both by RP column and by HILIC column.

**Fig. 5:**
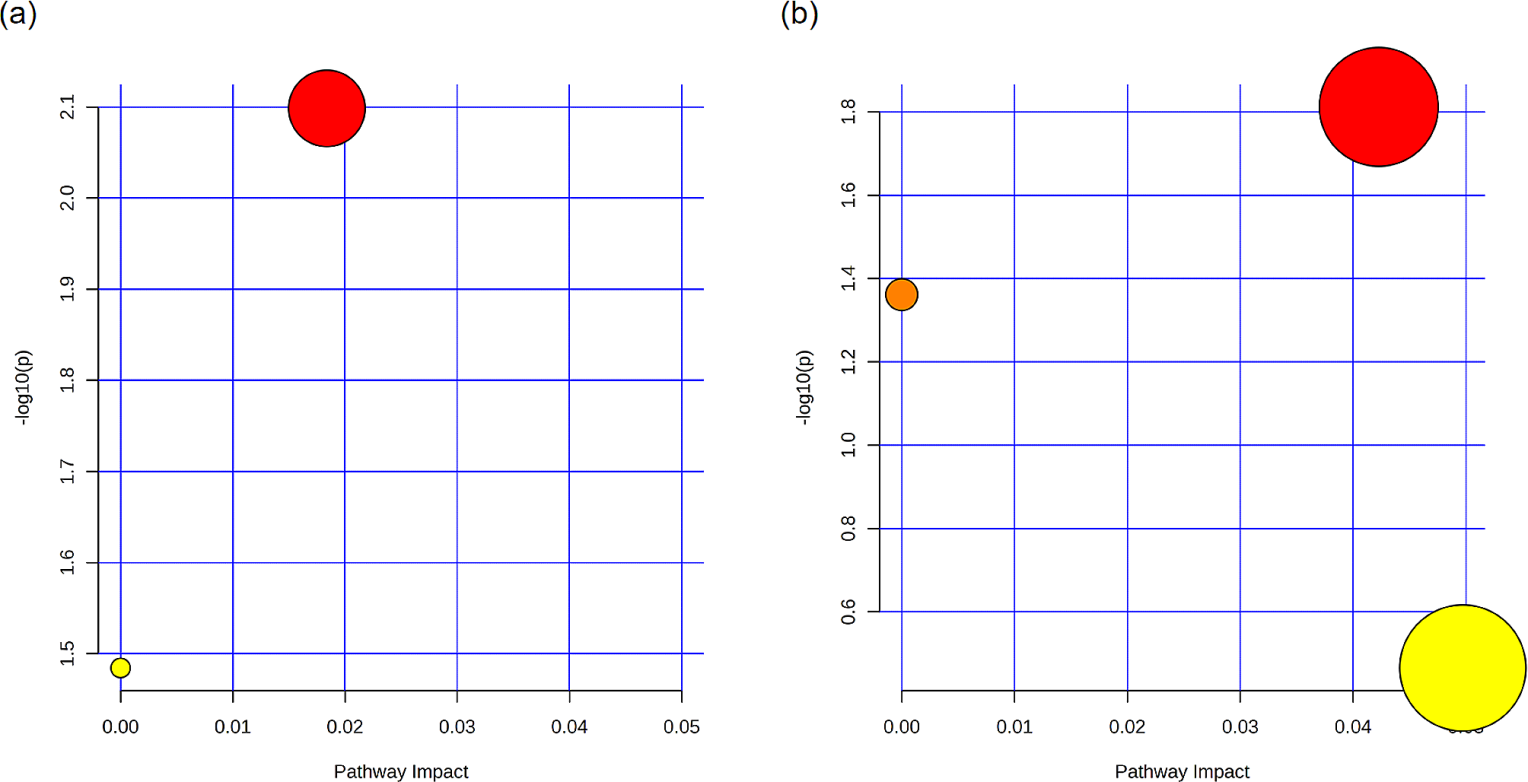
Metabolic pathway mapping in the KEGG database. The bubbles stand for KEGG pathways during early log phase (a) and during stationary phase (b), including vitamin B6 metabolism (orange), purine metabolism (yellow), and pyrimidine metabolism (red). The horizontal axis stands for the relative importance of metabolites in the pathways (Impact Value). The vertical axis stands for the significant enrichment significance of metabolites in the pathways (-log10(p)). The bubble size stands for the Impact Value. The larger the bubble, the greater the importance of the pathway

**Fig. 6:**
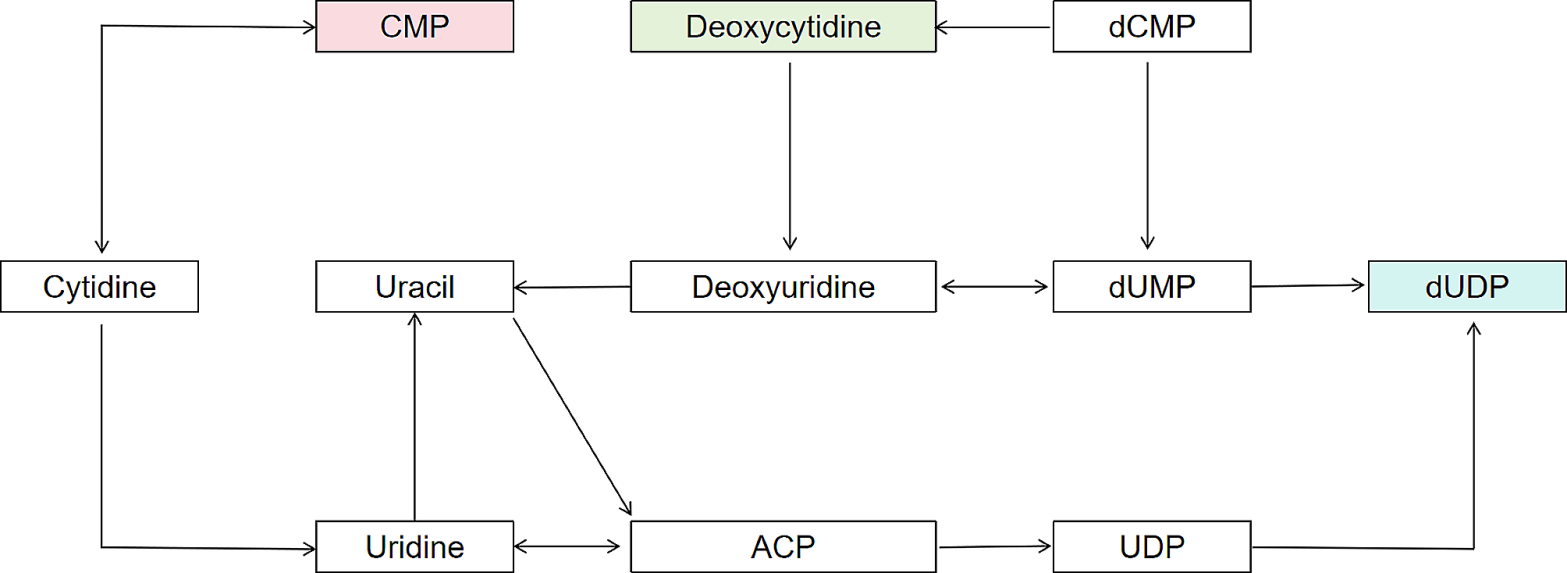
The schematic plot of the perturbed pyrimidine metabolism in *S. mutans* after fluoride-resistant mutation. Pyrimidine-related metabolite changes during early log phase (deoxycytidine, light green), stationary phase (dUDP, light blue), and both (CMP, light red). ACP, acyl-carrier protein; CMP, cytidine-5’-monophosphate; dCMP, deoxycytidine monophosphate; UDP, uridine pyrophosphate; dUDP, 2’-deoxyuridine 5’-diphosphate; dUMP, deoxyuridine monophosphate

Based on the assigned list of metabolites, pathway analysis was conducted using MetaboAnalyst 6.0. However, since the *S. mutans* pathway was not available in the program, *Streptococcus pyogenes* M1 476 (serotype M1) was utilized for the analysis. The principal metabolic pathways associated with these compounds included vitamin B6 metabolism, purine metabolism, and pyrimidine metabolism (Fig. 5). To validate these pathways for *S. mutans*, the KEGG database was consulted. In particular, the pyrimidine metabolism pathway for *S. mutans* revealed the release of deoxycytidine during early log phase and dUDP during stationary phase, aligning with the finding where CMP was among the distinct metabolites, irrespective of the growth phase (Fig. 6).

## Discussion

After the LC-MS metabolic analysis, the PLS-DA scores plot showed clear separation between *S. mutans* UA and *S. mutans* FR, suggesting its potential for identification of biological variations that occur in *S. mutans*. In our metabolomics study, it is a practical approach to separate *S. mutans* FR from *S. mutans* UA by LC-MS. The discriminant metabolites identified with *p* < 0.05 were validated with VIP (threshold set at 2.0). Successfully, 5 discriminant metabolites were identified in early log phase and 13 discriminant metabolites in stationary phase. Our results indicate that the metabolites from *S. mutans* FR may be related to the pathways of vitamin B6 metabolism (amino acid, carbohydrate, and fatty acid metabolism) and nucleotide metabolism (purine and pyrimidine metabolism).

### Carbohydrate metabolism

Fructose-1,6-bisphosphate (FBP) was identified as distinct markers from *S. mutans* in the carbohydrate metabolism pathway in this study. Acid production in the carbohydrate metabolism (the glycolysis process) is one of the virulent features of *S. mutans*. In comparison with the wild-type strains, *S. mutans* FR can produce more acid in the presence of fluoride [10, 23, 24]. However, in the absence of fluoride, *S. mutans* FR acid production rates are inconsistent and can be higher [10], lower [25], or the same [23]. In terms of acid tolerance, there are two controversial results. One shows *S. mutans* FR was more acid-sensitive, which could be more easily killed at a low pH value [6]. The other exhibits *S. mutans* FR had stronger ability to survive in acidified media than the wild-type strains [22].

FBP can activate phosphofructokinase (PFK) and pyruvate kinase in the glycolysis process and can promote the conversion of glucose to lactate [26]. Thus, the activity of lactate dehydrogenase in *Streptococcus* depends on the presence of FBP [27]. The fructose phosphotransferase system (PTS) transporters from *S. mutans* produce fructose-1-phosphate and fructose-6-phosphate, which can be converted into FBP by PFK [28].

In our study, we found that the FBP levels from fluoride-resistant *S. mutans* increased in the stationary phase compared to the levels from wild-type one. FBP concentration as a sensor of carbon influx was positively correlated with the growth rate [29]. FBP participates in the upstream process of glycolysis, as an important intermediate. It suggests the glycolysis of fluoride-resistant *S. mutans* might be more active than wild-type one. Further research includes the effect of FBP from fluoride-resistant strains on the glycolysis and why the acid-producing ability of fluoride-resistant strains enhances.

### Fatty acid metabolism

Fatty acids in cell membrane play a critical role in maintaining normal physiological function of cells. When cells are subjected to some environmental stress, such as temperature, ion, salt, drug, and oxidative stress, they can rapidly change their fatty acid composition in the cell membrane, alter their morphology, or increase mobility to resist the damage caused by the external environment [30, 31]. In cariogenic bacteria, the membranous fatty acids keep the acid-base balance inside and outside the cell membrane. At pH 5, the proportion of long chain monounsaturated fatty acids in the cell membrane of *S. mutans* is increased in response to the acid-resistant stress [32]. In our previous work, we also found that the amount of long chain monounsaturated fatty acids in the cell membrane of *S. mutans* increased under acidic conditions, and that the amount of monounsaturated fatty acids in *S. mutans* FR increased to a greater extent than the parental strains with the enhanced ability of acid resistance after *S. mutans* FR induced [22]. In this study, we found that the FBP level of fluoride-resistant strains was higher than that of wild-type ones under the same conditions.

It is widely accepted that FBP can affect the lipid metabolism [26]. It can activate acetyl coenzyme A carboxylase (acetyl CoA), and thereby can regulate lipid synthesis in yeast [33]. In addition, FBP can activate fatty acid synthetase in *E. coli*, which is probably because FBP increased the affinity of acetyl CoA in this bacterium, or because FBP promotes the stability of acetyl CoA [34].

Collectively, it is likely that the increased concentration of FBP in the fluoride resistant strains promotes unsaturated fatty acid synthase activity, and thus the amount of unsaturated fatty acids in fluoride-resistant strains increases. The exact interaction between FBP and fatty acid remains unclear. Further studies are warranted.

### Metabolic pathway of *S. mutans*

Currently, the database related to *S. mutans* metabolites has not yet been established. Since the study of *S. mutans* metabolomics is still at the exploratory stage, the potential biomarkers found in our study can only be speculated according to the database related to the metabolites of *Streptococcus pyogenes* M1 476 (serotype M1) (KEGG).

In our study, we found that there were many other different metabolites, for instance, pyridoxamine 5′-phosphate (PMP) and UDP, between fluoride-resistant strains and their parental strains. These presumably identified metabolites are generally related to bacterial vitamin B6 metabolism (amino acid, carbohydrate, and fatty acid metabolism) and nucleotide metabolism (purine and pyrimidine metabolism). Our results are consistent with previous studies. In amino acid metabolism pathway, *S. mutans* was inhibited by PMP acting on the glucosyltransferase [35]. UDP-associated metabolites [17] were reduced in arginine-treated (dental caries preventive agent treated) *S. mutans* from the nucleotide metabolism pathway view. Although the understanding of the detailed function of these metabolites is still limited, Eva-Maria Decker and colleagues reported that the exposure of *S. mutans* to xylitol resulted in distinct gene expression patterns. Specifically, *GtfC* exhibited upregulation exclusively in the presence of xylitol. Furthermore, under xylitol exposure, the upregulation of *gtfB* was sixfold, whereas under sucrose exposure, it was threefold [36]. Thus, the involved metabolic pathways of *S. mutans* FR in our study may be due to altered gene functions after the mutation of fluoride resistance.

Meanwhile, it is reported that Lysine lactylation, a posttranslational modification, affects bacterial survival in altered environments and pathogenicity by regulating energy metabolism and amino acid metabolism [37]. A study on the acetylome patterns of *S. mutans* reveals that the acetyltransferase ActA acetylated lactate dehydrogenase (LDH) and hindered LDH’s enzymatic capacity to facilitate the transformation of pyruvate into lactic acid, consequently diminishing its cariogenicity in a rat caries model [38]. The functional role of lactylation at Lys^173^ of RNA polymerase subunit α (RpoA) in *S. mutans* involves the regulation of exopolysaccharides synthesis in glycol-metabolic pathways [39]. In dental caries, therefore, the excess fluoride use may be responsible for the metabolic reprogramming to be more favorable for *S. mutans* FR associated with its acidogenicity and acid tolerance.

Systematic mapping of identified metabolites to metabolic pathways involves associating metabolites with the biochemical pathways in which they participate. By doing so, we can gain insights into the underlying biological processes that are influenced by the observed changes in metabolite concentrations. Recently, a genome-scale metabolic model for the UA159 strain, named iSMU, encompassing 675 reactions and incorporating 429 metabolites and the outputs of 493 genes, will pave the way to a comprehensive understanding of the metabolism of *S. mutans* [40]. Further research will probably reveal a largely unexplored facet of metabolomes toward the comprehensive coverage.

### Limitations

This study’s exclusive use of anaerobic cultivation for *S. mutans* does indeed prompt important considerations regarding the applicability of the findings to aerobic conditions in the oral environment. The metabolism of *S. mutans* can exhibit significant variations between anaerobic and aerobic environments due to differences in metabolic pathways, including available energy sources, oxygen sensitivity, and redox status. Specifically, *S. mutans* switches fermentation pathways towards oxidative phosphorylation to generate energy. This shift can lead to variations in metabolic intermediates, potentially impacting the levels of fructose-1,6-bisphosphate and other metabolites [26]. Understanding these differences in metabolic pathways between anaerobic and aerobic conditions is crucial for comprehending the physiological adaptations of *S. mutans.* This knowledge is valuable in the context of dental caries, where variations in the oral environment can impact the metabolic strategies employed by this bacterium.

Also, the single use of metabolomics technology may limit the identification of the distinguished markers between *S. mutans* UA and *S. mutans* FR. Due to a lack of a complete functional database for metabolomics, the unknown markers found in our study were not fully identified. Further research by multiple complementary analytical platforms needs to validate our results, such as transcriptomic analyses, DNA sequencing, and/or quantitative PCR.

## Conclusions

In summary, the elevated FBP concentration of *S. mutans* FR indicates its upgraded ability to produce acid and its enhanced capacity to tolerate acidic environment. This discriminant metabolite marker of *S. mutans* FR may lead to an applicable strategy to explore the cariogenic and fluoride resistant mechanisms.

### Electronic supplementary material

Below is the link to the electronic supplementary material.

Supplementary Material 1

Supplementary Material 2

## Data Availability

The raw data are available from the corresponding author upon reasonable request.

## Abbreviations

BHI: Brain Heart Infusion
ESI: Electrospray Ionization
FR: Fluoride-Resistant
FBP: Fructose-1,6-Bisphosphate
PTS: Fructose Phosphotransferase System
HILIC: Hydrophilic Interaction Liquid Chromatography
LC: Liquid Chromatography
MS: Mass Spectrometry
OSC: Orthogonal Signal Correction
PLS: DA-Partial Least-Squares Discriminant Analysis
PFK: Phosphofructokinase
PCA: Principal Component Analysis
PMP: Pyridoxamine 5′-Phosphate
RP: Reversed Phase
TIC: Total Ion Current
