# Supplementary material for: Metabolomics reveals high fructose-1,6-bisphosphate from fluoride-resistant Streptococcus mutans
Source: BMC Microbiol. 2024 May 3;24:151. doi: 10.1186/s12866-024-03310-8 (PMC11067228; doi:10.1186/s12866-024-03310-8)
Supplement: Supplementary file 1 — Supplementary Material 1 [file 12866_2024_3310_MOESM1_ESM.docx]

**Table S1** Modeling diagnostic of the metabolic data from cells of the fluoride-resistant strain and the wild-type strain in early log phase.

| Model^†^ | Column^‡^ | Component^§^ | Modeling diagnostic^¶^ | | |
| --- | --- | --- | --- | --- | --- |
|  |  |  | R^2^X | R^2^Y | Q^2^Y |
| PCA-X | RP | 2 | 0.579 | - | 0.360 |
|  | HILIC | 2 | 0.497 | - | 0.121 |
| PLS-DA | RP | 2 | 0.703 | 0.784 | 0.719 |
|  | HILIC | 2 | 0.523 | 0.891 | 0.793 |

^†^Model: PCA, principal component analysis; PLS-DA, partial least squares discriminant analysis.

^‡^Column: RP, reversed phase; HILIC, hydrophilic interaction liquid chromatography

^§^Component: 2 indicates quantities of components contributing to the cumulative values.

^¶^Modeling diagnostic: R^2^X, fraction of X-variation; R^2^Y, fraction of Y-variation; Q^2^Y, cumulated cross-validation for R^2^Y. Each value of R^2^X, R^2^Y and Q^2^Y ranges from 0 to 1.

**Table S2** Modeling diagnostic of the metabolic data from cells of the fluoride-resistant strain and the wild-type strain in stationary phase.

| Model^†^ | Column^‡^ | Component^§^ | Modeling diagnostic^¶^ | | |
| --- | --- | --- | --- | --- | --- |
|  |  |  | R^2^X | R^2^Y | Q^2^Y |
| PCA-X | RP | 3 | 0.667 | - | 0.030 |
|  | HILIC | 2 | 0.614 | - | 0.361 |
| PLS-DA | RP | 2 | 0.594 | 0.492 | 0.405 |
|  | HILIC | 2 | 0.661 | 0.499 | 0.425 |

^†^Model: PCA, principal component analysis; PLS-DA, partial least squares discriminant analysis.

^‡^Column: RP, reversed phase; HILIC, hydrophilic interaction liquid chromatography

^§^Component: 2 or 3 indicates quantities of components contributing to the cumulative values.

^¶^Modeling diagnostic: R^2^X, fraction of X-variation; R^2^Y, fraction of Y-variation; Q^2^Y, cumulated cross-validation for R^2^Y. Each value of R^2^X, R^2^Y and Q^2^Y ranges from 0 to 1.
