# Supplementary material for: Metabolomics reveals high fructose-1,6-bisphosphate from fluoride-resistant Streptococcus mutans
Source: BMC Microbiol. 2024 May 3;24:151. doi: 10.1186/s12866-024-03310-8 (PMC11067228; doi:10.1186/s12866-024-03310-8)
Supplement: Supplementary file 2 — Supplementary Material 2 [file 12866_2024_3310_MOESM2_ESM.docx]

**Supplementary figure**

**
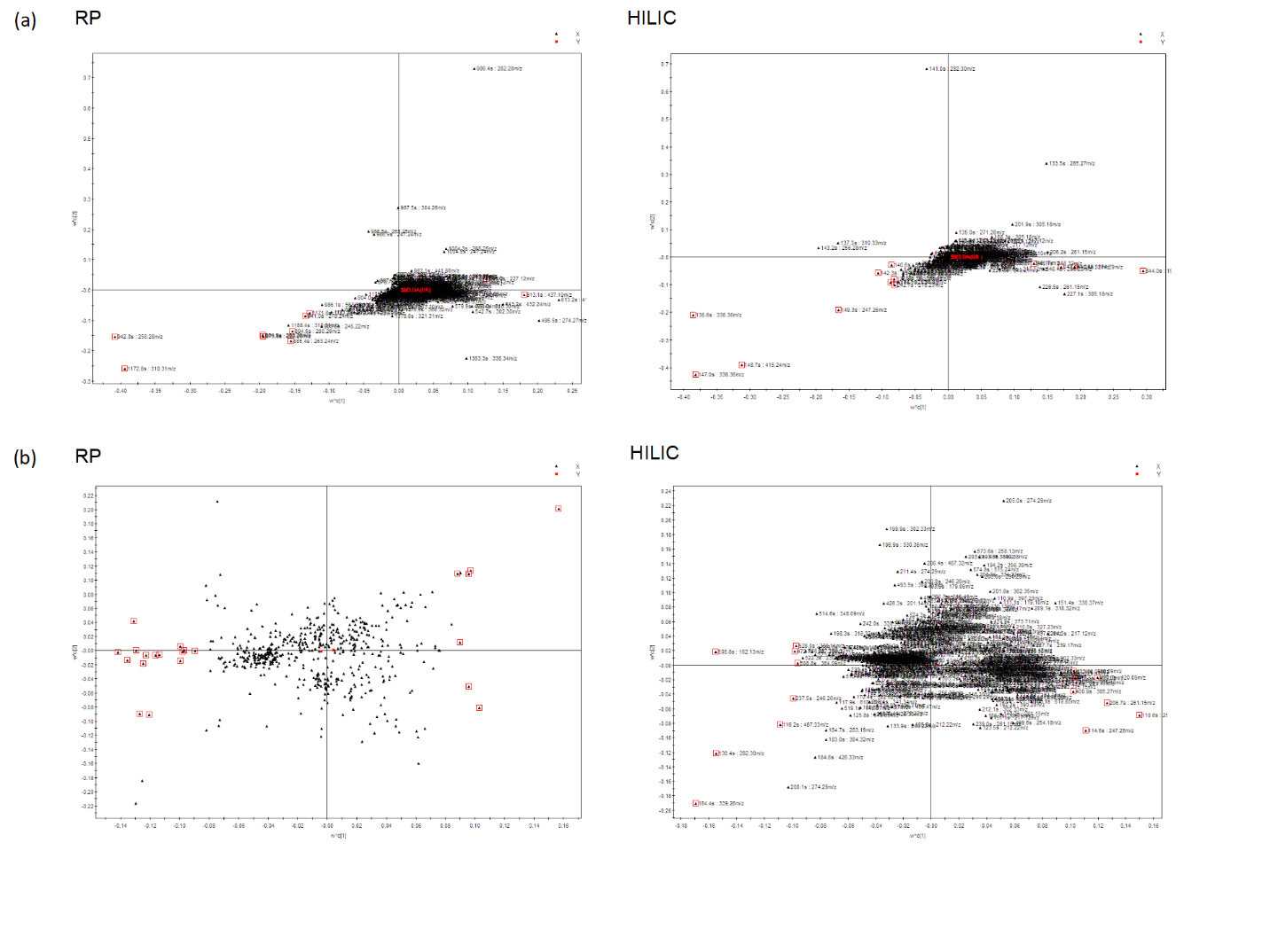
**

**Fig. S1**

Loading plot from PLS-DA model of LC-MS spectral data on *S. mutans*. (a) in early log phase; (b) in stationary phase. PLS-DA, partial least squares discriminant analysis; LC-MS, liquid chromatography-mass spectrometry; *S. mutans, Streptococcus mutans*; RP, reversed phase; HILIC, hydrophilic interaction liquid chromatography. UA represents wild-type (UA159) *S. mutans*. FR represents fluoride-resistant *S. mutans*.

**
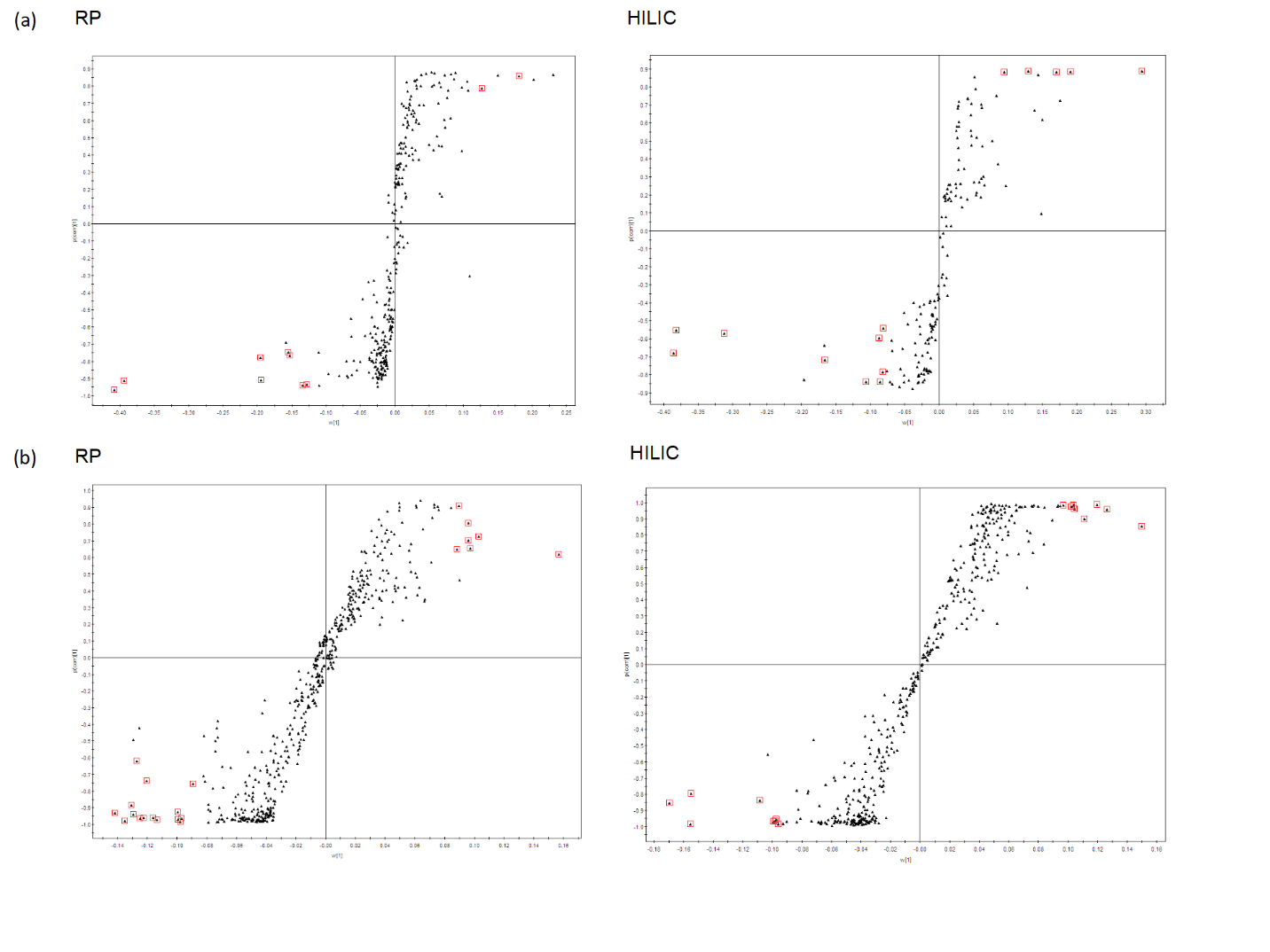
**

**Fig. S2**

S plot from PLS-DA model of LC-MS spectral data on *S. mutans*. (a) in early log phase; (b) in stationary phase. PLS-DA, partial least squares discriminant analysis; LC-MS, liquid chromatography-mass spectrometry; *S. mutans, Streptococcus mutans*; RP, reversed phase; HILIC, hydrophilic interaction liquid chromatography. *S. mutans* UA represents wild-type (UA159) *S. mutans*. FR represents fluoride-resistant *S. mutans*.
